# 

**Published:** 1895-09

**Authors:** 


					﻿ESTABLISHED 18551
SUBSCRIPTION, $2.00.
ATLANTA
JIEDIGfllt AND SURGlGflh
journalR'™^)
new serie”'. Atlanta, Ga., September, 1895. No. 7.
				

